# Probable cerebral amyloid angiopathy - related inflammation in a 32-year-old woman with down syndrome

**DOI:** 10.1007/s10048-026-00883-6

**Published:** 2026-02-12

**Authors:** Thales Pardini Fagundes, Gustavo Maximiano Alves, Mateus Gustavo Favaro, Joyce Yuri Silvestre Yamamoto, Lucas Ravagnani Silva, Lucas Giansante Abud, Jorge Alberto Martins Pentiado Junior

**Affiliations:** 1 Department of Head and Neck / Neurosurgery / Neuro-oncology, Hospital de Câncer de Barretos, Barretos, Brazil; 2 School of Medicine at Ribeirão Preto, Department of Neuroscience and Behavioural Sciences, Ribeirão Preto, Brazil; 3 Hospital São Lucas de Ribeirão Preto, Department of Diagnostic Imaging, Ribeirão Preto, Brazil; 4 Pain Center, Department of Neurology, University of São Paulo, São Paulo, Brazil; 5 Barretos Cancer Hospital, Barretos, São Paulo, Brazil

**Keywords:** Cerebral amyloid angiopathy, Down syndrome, Inflammation, Rituximab, Adult

## Abstract

Inflammatory cerebral amyloid angiopathy (CAA-RI) is a variant of CAA, rarely seen in young individuals with Down syndrome (DS). We describe a 32-year-old female with DS who developed a systemic inflammatory **state** followed by acute neurological symptoms, including seizures and hemiparesis. Brain MRI disclosed asymmetric T2/FLAIR hyperintensities with leptomeningeal enhancement and focal superficial siderosis on susceptibility-weighted imaging, without microbleeds; cerebrospinal fluid showed marked inflammation. After partial improvement with corticosteroids, rituximab was started due to persistent symptoms. This case highlights CAA-RI as a cause of acute decline in DS and supports the use of rituximab in refractory cases.

## Introduction

Cerebral amyloid angiopathy (CAA) is a common type of cerebral small vessel disease. It is characterized by amyloid-β (Aβ) deposition primarily affecting leptomeningeal and cortical vessels, often leading to intracerebral hemorrhage or cognitive decline in the elderly [1]. Within this spectrum lies CAA‑related inflammation (CAA‑RI), an immune reaction against vascular amyloid that presents with subacute deficits and imaging dominated by asymmetric vasogenic edema and leptomeningeal enhancement; microhemorrhagic markers may be absent early in the course [1].

By the fourth decade, virtually all adults with DS exhibit full Alzheimer-type neuropathology, and cerebral amyloid angiopathy (CAA) can manifest as early as the third decade [2]. Enlarged perivascular spaces and microinfarcts appear in the early 30s, while lobar microbleeds and white-matter injury emerge in the mid-to-late 30s. CAA is present in 31% of cognitively impaired and 13% of unimpaired adults with DS. APP locus triplication has resulted in symptomatic CAA at age 39, while partial trisomy 21 lacking APP triplication shows no CAA at autopsy, underscoring the gene-dosage effect [2–5].

This report describes a case of CAA-RI diagnosed in a 32-year-old female with Down syndrome, an age and genetic background where such inflammatory complications of CAA are seldom reported, particularly preceded by systemic inflammatory symptoms.

## Materials and methods

This is a descriptive case report. Clinical data, including medical history, physical examination findings, laboratory results, and neuroimaging studies, were collected from the patient’s electronic medical records at our institution.

## Results

The patient was a 32-year-old female with Down syndrome admitted with a systemic inflammatory state characterized by fever, vomiting, diarrhea, and dyspnea. According to the admission notes, she was febrile and tachypneic but not hypotensive; serial detailed vital signs were not consistently available for retrospective abstraction (Table 1).

Initial laboratory evaluation (Table 2 includes the patient’s laboratory and CSF results) during the systemic inflammatory phase (10–23 April 2023) showed a surge of inflammatory and reactive changes: erythrocyte sedimentation rate peaked at 135 mm/h, C-reactive protein peaked at 30 mg/L, leukocytosis reached 23,030/mm³, thrombocytosis peaked at 755,000/mm³, hemoglobin nadired at 9.3 g/dL, creatinine transiently rose to 1.77 mg/dL, with hyponatremia (131 mEq/L) and hypokalemia (3.0 mEq/L), followed by progressive normalization thereafter. Chest X-ray/CT and transthoracic echocardiography documented pleural and pericardial effusions. During the first week after admission, she was treated supportively, including antibiotics and diuretics, while an infectious and autoimmune work‑up was initiated; no CNS-directed immunosuppression was given before the onset of neurological symptoms.

**Table 1:** Auriel et al. (2016) diagnostic criteria vs. our patient

Auriel et al. (2016) criterion	Our patient	Evidence
Age > 40 years*	32 years	Exception justified by Down Syndrome
Subacute neurological symptoms	Yes	Acute onset with subacute progression
MRI: Asymmetric T2/FLAIR hyperintensities	Yes	Left hemisphere predominance
MRI: Leptomeningeal enhancement	Yes	Predominant in the left hemisphere
MRI: Cortical microbleeds (SWI)	No	No microbleeds; focal superficial siderosis present
CSF: Pleocytosis	Yes	150 cells/mm³ (73% lymphocytes)
CSF: Elevated protein	Yes	363 mg/dL
Exclusion of infections	Yes	Negative cultures
Exclusion of neoplasm	Yes	Negative cytology
FINAL DIAGNOSIS	**Probable CAA-related inflammation (CAA-RI)**	**Sensitivity 82% / Specificity 97%**

One week following admission, she developed neurological symptoms, including dysarthria, right-sided hemiparesis, altered mental status, and seizures. At presentation, the NIHSS score was 11 (1a = 1; 3 = 1; 4 = 2; 5b = 2; 6b = 2; 9 = 2; 10 = 1), Corresponding to a decreased level of consciousness, a visual field deficit, partial facial palsy, moderate weakness in the right arm and leg, severe aphasia, and mild dysarthria. Seizures were managed with Levetiracetam 1000 mg/day, with no recurrence. The modified Rankin Scale (mRS) was 4, compared with a pre-morbid mRS of 2 related to baseline functional dependence due to Down syndrome.

**Fig. 1:**
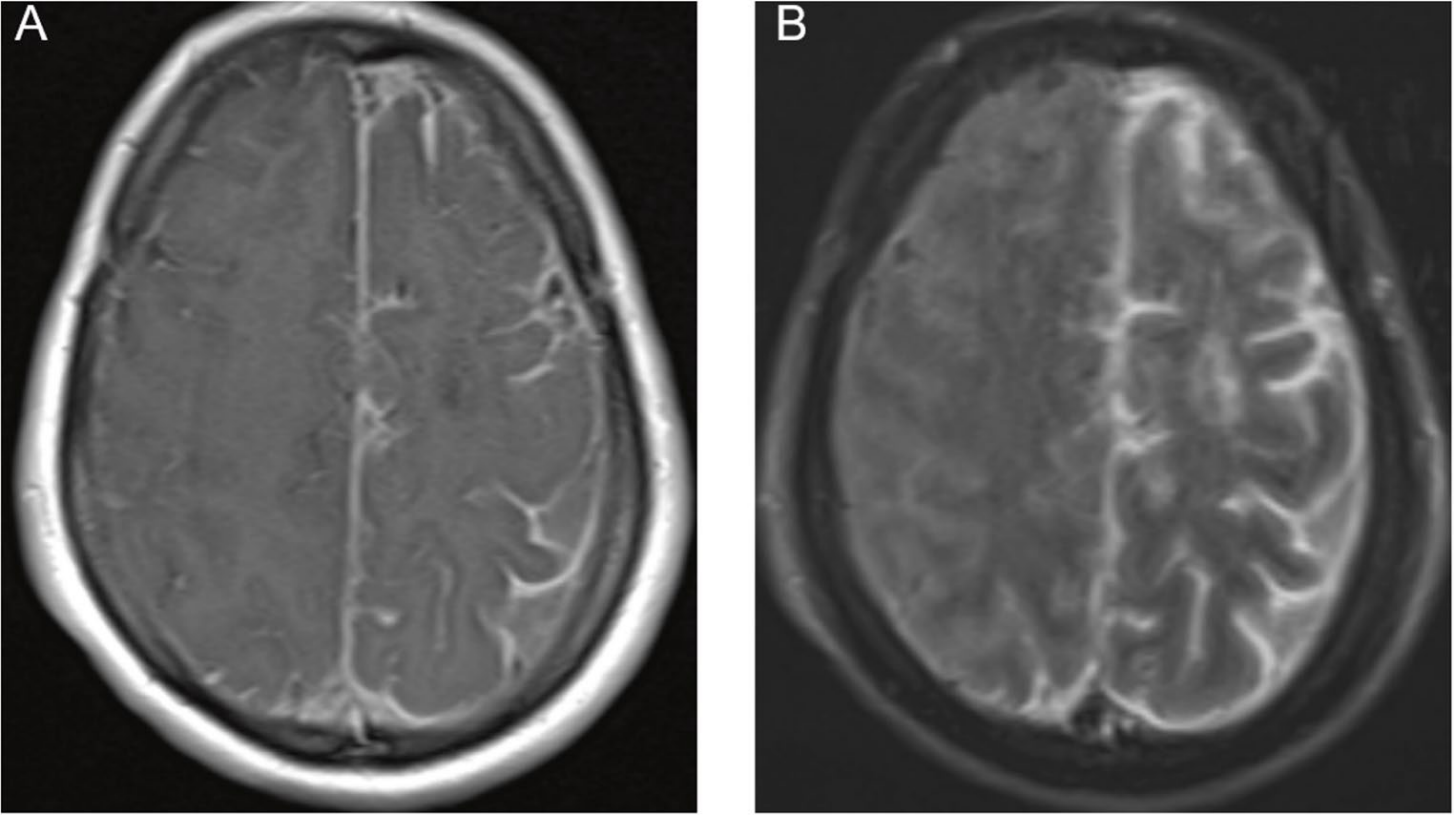
(**A**) Axial post‑contrast T1‑weighted MRI showing left‑predominant fronto‑parietal leptomeningeal enhancement following a gyral‑sulcal pattern and extending into superficial cortical vessels. (**B**) Axial post‑contrast FLAIR demonstrating the same topography with greater conspicuity and cortical/subcortical hyperintensity in the right superior and middle frontal gyri, consistent with vasogenic edema adjacent to the involved leptomeninges. Abbreviations: MRI = magnetic resonance imaging; FLAIR = fluid‑attenuated inversion recovery

**Fig. 2:**
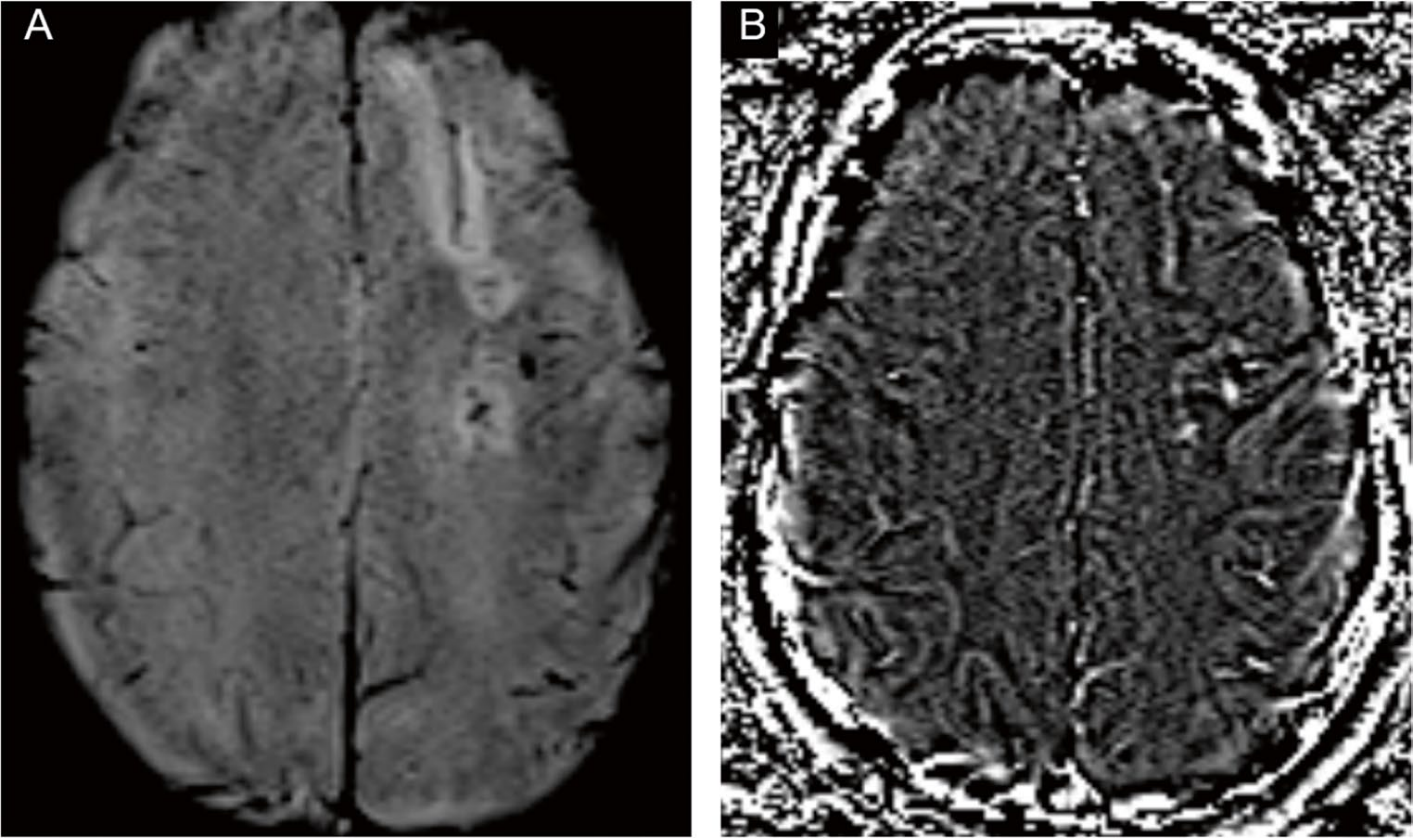
(**A**) Axial SWI magnitude image showing a linear hypointense rim along the left high frontal convexity, compatible with superficial siderosis. (**B**) Corresponding filtered-phase image with the same topography, confirming the paramagnetic nature of the deposits. No lobar or deep microbleeds are identified. Abbreviations: SWI = susceptibility-weighted imaging

**Fig. 3:**
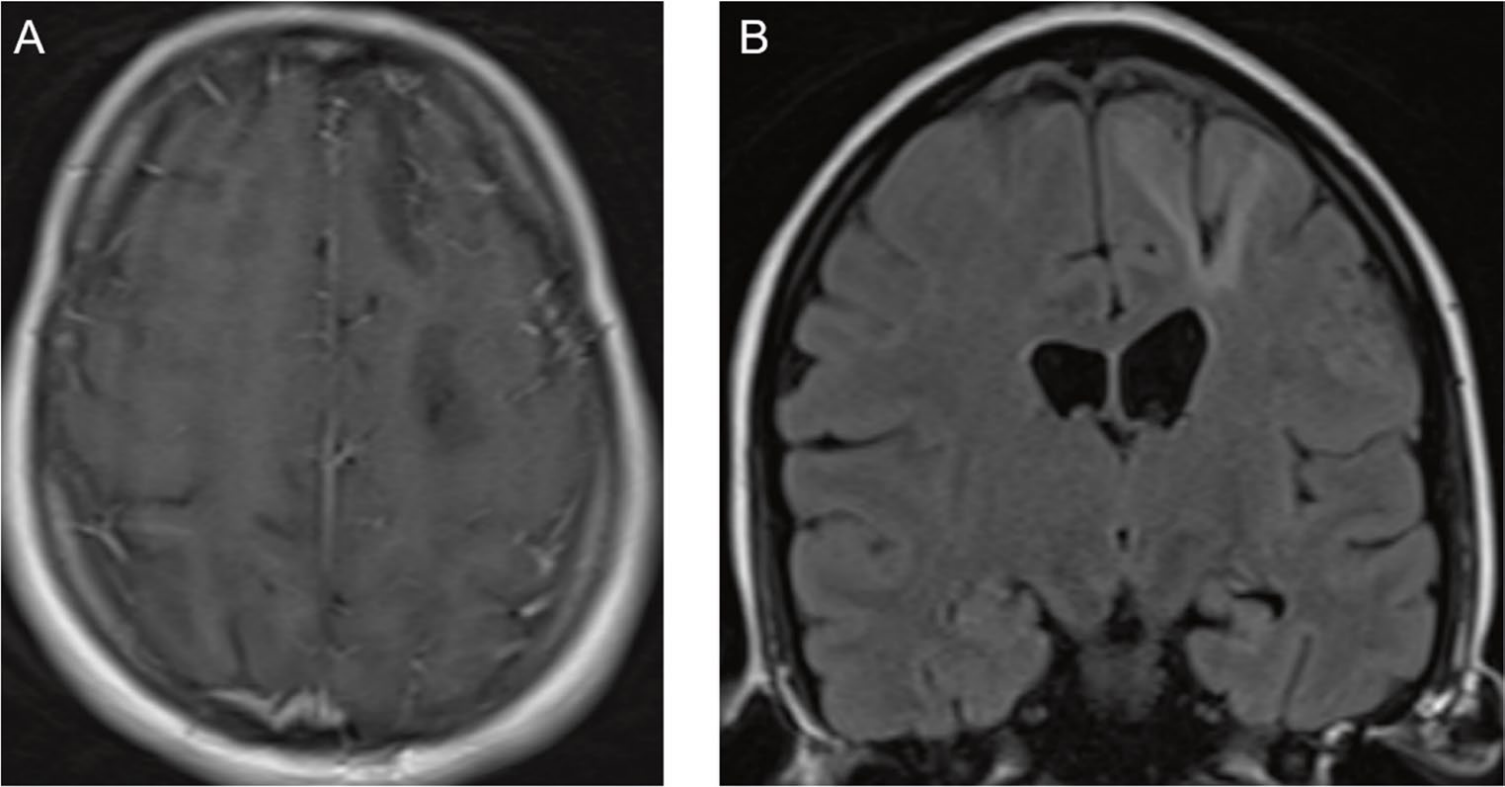
(**A**) Axial post‑contrast T1‑weighted MRI after corticosteroid therapy showing near‑complete regression of the previously observed leptomeningeal enhancement. (**B**) Coronal non‑contrast FLAIR demonstrating residual cortical/subcortical sequelae predominantly in the right superior and middle frontal gyri. Abbreviations: MRI = magnetic resonance imaging; FLAIR = fluid‑attenuated inversion recovery

Brain MRI demonstrated left‑predominant leptomeningeal enhancement over the fronto‑parietal convexities and cortical/subcortical T2‑FLAIR hyperintensity in the right superior and middle frontal gyri, consistent with vasogenic edema adjacent to the involved leptomeninges. Susceptibility-weighted imaging (SWI) acquired after contrast administration did not show microbleeds; a subsequent non‑contrast SWI disclosed a linear hypointense rim along the left high frontal convexity with a matching paramagnetic pattern on the filtered phase, compatible with focal superficial siderosis and still no microbleeds (Figs. 1 and 2). Taken together, these findings support CAA-RI and are illustrated in Figs. 1, 2, and 3. CSF analysis showed marked inflammation (protein 363 mg/dL, white blood cells 150/mm³, 73% lymphocytes). Gram stain and cultures were negative; cytology was negative. A multiplex PCR panel for neurotropic viruses (HSV‑1/2, VZV, CMV/EBV/HHV‑6, enterovirus, JC virus) was negative. Applying Auriel’s clinicoradiological criteria (2016), the patient fulfills probable CAA-RI: subacute neurological symptoms; asymmetric cortical/subcortical T2-FLAIR hyperintensity with leptomeningeal enhancement; SWI with focal superficial siderosis and no microbleeds; inflammatory CSF; exhaustive evaluation excluding infection and neoplasm. The age > 40 years threshold is exceeded biologically in Down syndrome, where accelerated vascular amyloid due to APP triplication advances the window for CAA and CAA-RI into the third decade; therefore, age < 40 does not preclude the diagnosis in this context.

Treatment consisted of intravenous methylprednisolone 1 g/day for five days followed by oral prednisone 60 mg/day tapered over six months. Clinical improvement was partial, with persistent systemic activity and residual radiological inflammation; therefore, rituximab 375 mg/m² was administered intravenously once weekly for four consecutive weeks (July 2023), with no relapses thereafter. This regimen was extrapolated from CNS vasculitis and refractory CAA-RI cohorts and resulted in an improvement in the NIHSS scale to 0, and a return to mRs 2 (baseline) after the final July 2023 pulse [6].

## Discussion

This report describes inflammatory cerebral amyloid angiopathy (CAA-RI) in a 32-year-old woman with Down syndrome (DS), an unusually early age of onset compared with the typical mean of 67 years [1]. While cerebral amyloid angiopathy (CAA) is common in DS due to amyloid precursor protein (APP) gene triplication [7], the inflammatory subtype is rare. Neuropathological and microglial data in DS further support a pro-inflammatory milieu around vascular amyloid. Published biomarker studies in DS (including sTREM2 and cytokine signatures) provide a plausible substrate for such susceptibility, even though the studies in our report are not patient-derived [8–15]. This framework explains the early onset (32 years) of CAA-RI despite the > 40-years age threshold in the general criteria and aligns with the radiological evolution in which susceptibility markers may be absent at onset and become apparent later.

The sequence in this patient, systemic inflammatory flare followed by subacute neurological deficits with asymmetric edema and leptomeningeal enhancement, raises a pathophysiological link in Down syndrome, where innate immune dysregulation lowers the threshold for CNS inflammation [12–14]. However, temporal association alone does not establish causality, and alternative or concurrent triggers cannot be excluded. The subsequent acute neurological deficits, including hemiparesis and seizures, align with common CAA-RI presentations such as cognitive decline and headache [1, 10–12].

The diagnosis was supported by characteristic neuroimaging and cerebrospinal fluid (CSF) analysis. The patient met criteria for probable CAA-RI. Table 1 compares Auriel et al.‘s (2016) diagnostic criteria with our patient. Brain MRI disclosed asymmetric T2/FLAIR hyperintensities with leptomeningeal enhancement and focal superficial siderosis on susceptibility-weighted imaging, without microbleeds; cerebrospinal fluid showed marked inflammation. Although microbleeds are frequent markers of underlying CAA [1, 8, 16, 17], their absence does not exclude CAA-RI early in the disease, particularly when the clinicoradiological constellation is otherwise typical. The CSF showed marked inflammation, with high protein and pleocytosis, consistent with over 80% of CAA-RI cases [6, 18]. Although not performed here, the *APOE ε4/ε4* genotype is a significant risk factor for CAA-RI, and CSF Aβ levels may be altered [3]. Anti-amyloid-β autoantibodies were not assessed in this patient.

**Table 2:** Diagnostic work‑up

Domain	Test	Result
CSF - cell/protein	WBC and differential; Protein; Glucose	150/mm³ (73% lymphocytes); 363 mg/dL; glucose exceeds two-thirds of serum level
CSF - microbiology	Gram stain and cultures; AFB stain/culture	Negative
CSF - oncology	Cytology (± flow cytometry)	Negative
CSF - PCR panel	HSV-1/2, VZV, CMV/EBV/HHV-6, enterovirus, JC virus	Negative
Serology	HIV, syphilis, hepatitis panel	Negative
Autoimmune/systemic	ANA/ENA, ANCA, C3/C4, thyroid panel, ESR/CRP	ESR/CRP No evidence of systemic autoimmune disease; ESR peaked at 135 mm/h; CRP 30 mg/L
Systemic imaging	Chest X-ray/CT; echocardiography	Pleural and pericardial effusions

Although the Auriel criteria typically require age > 40 years for the diagnosis of CAA-RI, Down syndrome represents a well-recognized exception due to its accelerated amyloid pathology. Neuropathological studies have demonstrated that virtually all individuals with Down syndrome develop full Alzheimer’s disease pathology by the fourth decade, with cerebral amyloid angiopathy emerging as early as the third decade [2]. Microbleeds and vascular amyloid deposition appear in the mid-to-late thirties, significantly earlier than in the general population [19].

Our patient, aged 32 years, falls within this expected window for CAA-related complications in Down syndrome. The triplication of the APP gene on chromosome 21 results in lifelong amyloid-β overproduction [7], making Down syndrome one of the strongest genetic risk factors for early-onset CAA. A case of APP locus triplication with CAA onset at 39 years has been reported [2], only seven years older than our patient. These observations support adapting CAA-RI diagnostic criteria for Down syndrome, with age thresholds adjusted to account for the accelerated timeline of amyloid deposition characteristic of this population.

Brain biopsy was deferred given fulfillment of probable CAA-RI with high-specificity clinicoradiological criteria, typical MRI and inflammatory CSF, exhaustive negative infectious/neoplastic evaluations, and a rapid response to immunosuppression; biopsy was reserved for atypical evolution or treatment failure.

The treatment course reflects the variable response of CAA-RI to immunosuppression. High-dose corticosteroids are first-line therapy, and immunosuppressive treatment is associated with better clinical outcomes and fewer recurrences [6, 7, 20]. In this case of partial steroid response, rituximab was used, a strategy supported by observational data for maintenance therapy in refractory CAA-RI [21, 22]. Patients with the Aβ-related angiitis (ABRA) subtype may require combination therapy more often, though relapse rates can remain high compared to primary angiitis of the central nervous system (PACNS) [21].

The primary limitation is the lack of neuropathological confirmation. The diagnosis of probable CAA-RI was based on clinicoradiological criteria with high sensitivity and specificity [23], but a brain biopsy was not performed. As a single case report, these findings are not generalizable to the heterogeneous DS population.

This case shows that, in Down syndrome, accelerated vascular amyloid makes age < 40 an inadequate exclusion for CAA‑RI; when clinicoradiological criteria are met, CAA‑RI should be considered irrespective of age. It highlights a potential interaction between the early amyloid pathology in DS and the population’s unique neuroinflammatory state [15, 24]. Future prospective studies using advanced biomarkers are needed to better define the incidence, triggers, and optimal management of CAA-RI in this specific population.

## Conclusion

Immunomodulatory treatments can improve clinical and radiological outcomes in patients with CAA-RI and reduce relapse risk. However, data remain limited due to the rarity of this disorder. Further research is needed to understand its underlying pathophysiological mechanisms better, refine therapeutic approaches, and optimize long-term patient outcomes.

## Data Availability

No datasets were generated or analysed during the current study.

## References

[1] Wu J-J, Yao M, Ni J (2021) Cerebral amyloid angiopathy-related inflammation: current status and f Uture implications. Chin Med J 134(6):646–654. 10.1097/cm9.0000000000001427 33625036 10.1097/CM9.0000000000001427 PMC7990003

[2] Grangeon L, Cassinari K, Rousseau S, Croisile B, Formaglio M, Moreaud O et al (2021) Early-Onset cerebral amyloid angiopathy and alzheimer disease related to an APP locus triplication. Neurol Genet 7(5):e609. 10.1212/nxg.0000000000000609 34532568 10.1212/NXG.0000000000000609 PMC8439959

[3] Carmona-Iragui M, Balasa M, Benejam B, Alcolea D, Fernandez S, Videla L et al (2017) Cerebral amyloid angiopathy in down syndrome and sporadic and autosomal-dominant alzheimer’s disease. Alzheimers Dement 13(11):1251–1260. 10.1016/j.jalz.2017.03.007 28463681 10.1016/j.jalz.2017.03.007 PMC5660938

[4] Patrick JL, Natalie CE, Lisi F-A, Mohamad JA, Batool R (2024) Cerebrovascular disease emerges with age and alzheimer’s disease in ad Ults with down syndrome. Sci Rep. 10.1038/s41598-024-61962-y39414925

[5] Doran E, Keator D, Head E, Phelan MJ, Kim R, Totoiu M et al (2017) Down Syndrome, partial trisomy 21, and absence of alzheimer’s disease: the role of APP. J Alzheimers Dis 56(2):459–470. 10.3233/jad-160836 27983553 10.3233/JAD-160836 PMC5662115

[6] Regenhardt RW, Thon JM, Das AS, Thon OR, Charidimou A, Viswanathan A et al (2020) Association between immunosuppressive treatment and outcomes of cerebral amyloid angiopathy–related inflammation. JAMA Neurol 77(10):1261–1269 32568365 10.1001/jamaneurol.2020.1782 PMC7309570

[7] Lott IT, Head E (2019) Dementia in down syndrome: unique insights for alzheimer disease Resea Rch. Nat Reviews Neurol 15(3):135–147. 10.1038/s41582-018-0132-6 10.1038/s41582-018-0132-6 PMC8061428 30733618

[8] Aranha MR, Fortea J, Carmona-Iragui M (2022) Cerebral amyloid Angiopathy–Related inflammation in down Syndrome–Rela Ted alzheimer disease. Neurology 98(24):1021–1022. 10.1212/wnl.0000000000200704 35470138 10.1212/WNL.0000000000200704 PMC9231835

[9] Belza M (1986) H, U. Cerebral amyloid angiopathy in Down’s syndrome. Clinical Neuropathology. Available from 2949903

[10] Naito K-S, Sekijima Y, Ikeda S-I (2008) Cerebral amyloid angiopathy-related hemorrhage in a middle-aged Patien t with down’s syndrome. Amyloid 15(4):275–277. 10.1080/13506120802524981 19065301 10.1080/13506120802524981

[11] Romero Lopez J, Rivas Infante E, Macineiras Montero JL, Carretero M (2006) Angiopatia Amiloidea cerebral, hemorragias cerebrales de repeticion y sindrome de down [Cerebral amyloid angiopathy, recurrent intracerebral haemorrhages and down’s syndrome]. Neurologia 21(10):729–732 Available from 17106827

[12] McCarron MO, Nicoll JAR, Graham DI (1998) A quartet of down’s syndrome, alzheimer’s disease, cerebral amyloid an giopathy, and cerebral haemorrhage: interacting genetic risk factors. J Neurol Neurosurg Psychiatry 65(3):405–406. 10.1136/jnnp.65.3.405 9728967 10.1136/jnnp.65.3.405 PMC2170259

[13] Iulita MF, Ower A, Barone C, Pentz R, Gubert P, Romano C et al (2016) An inflammatory and trophic disconnect biomarker profile revealed in D own syndrome plasma: relation to cognitive decline and longitudinal Ev Aluation. Alzheimer’s Dement 12(11):1132–1148. 10.1016/j.jalz.2016.05.001 27452424 10.1016/j.jalz.2016.05.001

[14] Weber GE, Koenig K, Khrestian M, Shao Y, Tuason ED, Gramm M et al (2019) Altered relationship between soluble TREM2 and inflammatory markers in young adults with down syndrome. bioRxiv 10.1101/776583 10.4049/jimmunol.1901166 PMC7033027 31959733

[15] Koenig KA, Ruedrich S, Bekris LM, Khrestian M, Kim S, Leverenz JB (2019) P3-065: White matter integrity and inflammation at 7 tesla in adults with down syndrome. Alzheimer’s Dement 15(7SPart18). 10.1016/j.jalz.2019.06.3092

[16] Kinnecom C, Lev M, Wendell L, Smith E, Rosand J, Frosch M et al (2007) Course of cerebral amyloid angiopathy–related inflammation. Neurology 68(17):1411–1416 Available from 17452586 10.1212/01.wnl.0000260066.98681.2e

[17] Helman AM, Siever M, McCarty KL, Lott IT, Doran E, Abner EL et al (2019) Microbleeds and cerebral amyloid angiopathy in the brains of people with down syndrome with alzheimer’s disease. J Alzheimers Dis 67(1):103–112. 10.3233/jad-180589 30452414 10.3233/JAD-180589 PMC6424116

[18] Corovic A, Kelly S, Markus HS (2018) Cerebral amyloid angiopathy associated with inflammation: a systematic review of clinical and imaging features and outcome. Int J Stroke 13(3):257–267 Available from 29134927 10.1177/1747493017741569

[19] Patrick JL, Amirreza S, Natalie CE, Rafael VL, Batool R, Anna CS et al (2023) Pseudo-longitudinal trajectories of cerebrovascular disease biomarkers in adults with down syndrome across the lifespan. Alzheimer’s Dement. 10.1002/alz.079407

[20] Eng JA, Frosch MP, Choi K, Rebeck GW, Greenberg SM (2004) Clinical manifestations of cerebral amyloid angiopathy–related inflammation. Ann Neurol 55(2):250–256. 10.1002/ana.10810 14755729 10.1002/ana.10810

[21] Hoshina Y, Delic A, Wong K-H, Lyden S, Kadish R, Smith TL et al (2023) Vasculitis in the central nervous system: Etiology, Characteristics, and outcomes in a large Single-Center cohort. Neurohospitalist 14(2):129–139. 10.1177/19418744231223283 38666288 10.1177/19418744231223283 PMC11040621

[22] Alokley AA, Alshamrani FJ, Alabbas FM, Nazish S (2021) When brain biopsy solves the dilemma of diagnosing atypical cerebral Amyoild angiopathy: A case report. Am J Case Rep 22e933869. 10.12659/ajcr.933869 10.12659/AJCR.933869 PMC8579063 34735418

[23] Auriel E, Charidimou A, Gurol ME, Ni J, Van Etten ES, Martinez-Ramirez S et al (2016) Validation of clinicoradiological criteria for the diagnosis of cerebr al amyloid Angiopathy–Related inflammation. JAMA Neurol 73(2):197. 10.1001/jamaneurol.2015.4078 26720093 10.1001/jamaneurol.2015.4078

[24] Wilcock DM (2012) Neuroinflammation in the aging down syndrome brain; lessons from alzheimer’s disease. Curr Gerontol Geriatr Res 20121–20110. 10.1155/2012/170276 10.1155/2012/170276 PMC3290800 22454637

